# Precise Mapping of Otp Expressing Cells Across Different Pallial Regions Throughout Ontogenesis Using Otp-Specific Reporter Transgenic Mice

**DOI:** 10.3389/fncir.2022.831074

**Published:** 2022-02-17

**Authors:** Lorena Morales, Alba González-Alonso, Ester Desfilis, Loreta Medina

**Affiliations:** ^1^Departament de Medicina Experimental, Universitat de Lleida, Lleida, Spain; ^2^Laboratory of Evolutionary Developmental Neurobiology, Lleida’s Institute for Biomedical Research Dr. Pifarré Foundation (IRBLleida), Lleida, Spain; ^3^Serra Húnter Fellows, Lleida, Spain

**Keywords:** pallium, basomedial amygdala, cortical amygdala, cingulate cortex, cingulate bundle, frontal cortex, hippocampus, parahippocampal lobe

## Abstract

Taking advantage of two Otp-specific reporter lines of transgenic mice (Otp-eGFP and Otp-Cre; Rpl22-HA), we identify and describe different Otp cell populations across various pallial regions, including the pallial amygdala, the piriform cortex, the mesocortex, the neocortex, and the hippocampal complex. Some of these populations can be followed throughout development, suggesting migration from external sources (for example, those of the pallial amygdala and at least some of the cingulate cortex). Other cells become visible during postnatal development (some of those in the neocortex and hippocampal formation) or in adulthood (those of the parahippocampal lobe), and seem to be produced locally. We discuss the possible role of Otp in these different populations during different moments of ontogenesis. We also analyze the connectivity patterns of some of these cells and discuss their functional implications. For example, our data suggest that Otp cells of the pallial amygdala might be engaged in networks with other Otp cells of the medial amygdala with the same embryonic origin, and may regulate specific aspects of social behavior. Regarding Otp cells in the parahippocampal lobe, they seem to be projection neurons and may regulate hippocampal function during spatial navigation and memory formation. The two reporter transgenic mice employed here provide very powerful tools for high precision studies on these different Otp cells of the pallium, but careful attention should be paid to the age and to differences between lines.

## Introduction

Neuron classification across cerebral cortex and other parts of the brain is undergoing a revolution thanks to the incorporation of powerful data from single cell transcriptomics, proteomics, genomics and epigenomics, which allow high-resolution analysis of cells across different areas, and explain how they originate and function in relation to landscapes that dynamically change throughout ontogenesis or in disease (Trapnell, 2015; Tasic et al., 2018; Yuste et al., 2020; Armand et al., 2021; Zhang et al., 2021). This approach is also helping to better understand the evolution of different cell types by discerning between conservation, divergence, and convergence processes (Tosches et al., 2018; Yuste et al., 2020; Colquitt et al., 2021). Some of these studies have provided insightful conclusions when using transcription factors vs. activity-dependent proteins. It appears that transcription factors are the most robust discriminators of different cell types and their progenitors during development (reviewed by Ruiz-Ortiz and Tollkuhn, 2021) and, when analyzed in combination and within the brain topological framework, they constitute a better reference to identify evolutionarily conserved cells (discussed by Medina et al., 2021). Cell-type specific genetic tools using reporter transgenic animals are also very powerful to gain access to individual neuron populations for high precision studies on their distribution, morphology, molecular features, connectivity, and function (Daigle et al., 2018).

Studies using this approach are still in their infancy, but they are providing enormous amounts of data that are helping to identify new cell types. For identification of cell types, in addition to publications, another important source of information comes from open access platforms that display raw or partially analyzed data on gene expression in the brain and other tissues in mouse, human and other species (for example, the web of the Allen Developing Mouse Brain Atlas^1^; or that of the Human Protein Atlas^2^). The Human Protein Atlas obtains data using various omics technologies (such as RNA-seq) and has provided evidence of scarce, but strong mRNA expression of the transcription factor Orthopedia (Otp) in the cerebral cortex of pig, mouse and human^3^. Otp is a transcription factor previously shown to be involved in the migration and differentiation of hypothalamic neuroendocrine neurons (Acampora et al., 1999, 2000; Wang and Lufkin, 2000) and medial extended amygdala neurons (García-Moreno et al., 2010). According to the Human Protein Atlas, part of the Otp expression occurs in neurons, pointing to the existence of previously unnoticed cell type (s) or subtype (s) in the cerebral cortex. The aim of our study was to further investigate the Otp cells of the pallium by taking advantage of two Otp-specific reporter lines of transgenic mice: Otp-eGFP and Otp-Cre; Rpl22-HA. In the Otp-eGFP, once the protein GFP is produced, it remains highly stable in the cytoplasm and processes of Otp cells, and is seen even after downregulation of Otp expression. This allowed analysis of cell morphology and axonal projections throughout ontogenesis (from embryos to adults). In the Otp-Cre; Rpl22-HA, the tag expression is related to a hemagglutinin (HA) tail in the ribosomal protein RPL22 (Rpl22-HA) that is Cre-dependent (Sanz et al., 2019), being in this case associated to Otp cells. The latter transgenic mouse was used for confirmatory purposes on the presence of Otp cells across different areas of the pallium.

## Materials and Methods

### Mice

In the present study, we employed two different Otp-specific reporter mice: (1) Otp-eGFP transgenic mice (*Mus musculus*, Tg (Otp-EGFP) OI121Gsat/Mmucd; Mutant Mouse Resource and Research Centers, MMRRC supported by NIH, University of California at Davis, United States), from 12.5 days of embryonic development (post-coitum; E12.5) to postnatal day 19 (P19), and adults (from 100 to 337 days) (21 embryos; 7 postnatal P6 to P19; 16 adults). (2) Otp-Cre; Rpl22-HA double transgenic mice (6 adults: P60 and P100), obtained by crossing a mouse female Rpl22-HA (*Mus musculus*, RiboTag mouse line (Sanz et al., 2009, 2019), kindly provided by Dr. Elisenda Sanz, Universitat Autònoma de Barcelona) with a mouse male Otp-Cre (*Mus musculus*, ordered to Cyagen Biosciences Inc., Santa Clara, CA, United States). The genotype and sex of animals was determined by PCR (McClive and Sinclair, 2001) at the Proteomics and Genomics Service of the University of Lleida. These transgenic mice lines were kept in the pathogen-free area of the rodent animal Facility of the University of Lleida (REGA license no. ES251200037660), which fulfills all requirements for genetically modified animals (notification no. A/ES/19/I-06). Progenitors and weaned-off postnatal animals were housed in groups of three to five at 22 ± 2°C on a 12-h light/dark cycle, with food and water *ad libitum*. All the animals were treated according to the regulations and laws of the European Union (Directive 2010/63/EU) and the Spanish Government (Royal Decree 53/2013) for the care and handling of animals in research. All the protocols used were approved by the Committees of Ethics for Animal Experimentation and Biosecurity of the University of Lleida and the Catalonian Government (Generalitat de Catalunya).

### Tissue Collection and Fixation

At appropriate development days, the mouse embryos were obtained by cesarean section from pregnant females, which were previously sacrificed by a lethal dose of sodium pentobarbital (0.1 mg/g; i.p.). Upon extraction, early embryos (E12.5-E14.5) were rapidly sacrificed by decapitation. The brains were dissected and fixed by immersion in phosphate-buffered 4% paraformaldehyde (4% PFA; pH7.4; 0.1 M) overnight at 4°C. Older embryos and postnatal animals (E16.5 to adults) were deeply anesthetized with sodium pentobarbital (0.1 mg/g; i.p.) and then transcardially perfused with 0.9% saline solution (0.9% NaCl), followed by 4% PFA. After dissection, the brains were postfixed by immersion in 4% PFA overnight at 4°C.

### Sample Preparation and Sectioning

Embryonic Otp-eGFP brains (E12.5-E18.5) and P100 Otp-Cre; Rpl22-HA brains were embedded in 4% low-melt agarose (LOW EEO, Laboratorios Conda S.A., Spain) in saline phosphate buffer (PBS; pH = 7.4) and sectioned using a vibratome (Leica VT 1000S, Leica Microsystems GmbH, Germany) in frontal, sagittal or horizontal plane, at 80 μm of thickness.

Postnatal Otp-eGFP brains and P60 Otp-Cre; Rpl22-HA brains were cryoprotected by immersion in a solution of glycerol and DMSO in PB [following the protocol of Rosene et al. (1986)], frozen with −60–70°C isopentane (2-methyl butane, Sigma-Aldrich, Germany) combined with dry ice for about 1 min and preserved at −80°C until use. Frontal free-floating sections, 40–60 μm-thick, were obtained using a freezing microtome (Microm HM 450, Thermo Fisher Scientific, United Kingdom), collected in cold PBS and processed as explained next.

### Immunohistochemistry to Detect Either GFP or HA

Free-floating vibratome and microtome sections were permeabilized by washing 3 times for 10 min with PBS containing 0.3% Triton X-100 (PBS-Tx; pH 7.4), followed by an incubation with a blocking solution composed of 10% normal goat serum (NGS) and 2% of bovine serum albumin (BSA) in PBS-Tx for 1 h at room temperature (RT). Subsequently, the sections were incubated in either primary chicken anti-GFP antibody or primary rabbit anti-HA antibody, as appropriate, diluted in PBS-Tx (see Table 1), for 72 h at 4°C. After washings in phosphate buffered saline (PBS), the endogenous peroxidase activity was reduced by an incubation in PBS containing 1% H_2_O_2_ and 10% methanol for 30 min. Then, the sections were washed and incubated in the proper biotinylated secondary antibody (see Table 2), diluted in PBS-Tx, for 90–120 min at RT. Next, the sections were washed and then incubated with the avidin-biotin complex (AB Complex, Vector Laboratories Ltd.) for 1 h at RT. After abundant washing, the sections were stained with a Tris buffered solution (0.05 M, pH 7.6), containing diaminobenzidine (DAB), urea and H_2_O_2_ for a few minutes until the proper intensity was obtained. Finally, the sections were washed, mounted using a solution of Tris buffer containing 0.25% gelatin, dehydrated and coverslipped with Permount (Thermo Fisher Scientific, United Kingdom).

**TABLE 1:** Primary antibodies.

Primary antibodies						
**Type**	**Antibody**	**Antigen recognized**	**Immunogen**	**Dilution**	**Manufacturer and Reference**	**RRID**

Polyclonal	Chicken anti-GFP, IgY	Green fluorescent protein (GFP)	Recombinant full-length protein corresponding to GFP	1:1,000	Abcam Antibodies, Ref. ab13970	AB_300798
	Rabbit anti-GFP, IgG	Green fluorescent protein (GFP)	Fusion protein corresponding to the full length GFP (246 aa)	1:1,000	Novus Biologicals, Ref. NB600-308	AB_10003058
	Rabbit anti-HA.11, IgG	Hemagglutinin	Twelve amino acid sequence CYPYDVPDYASL derived from the human influenza hemagglutinin surface glycoprotein (98–106 aa)	1:1,000	Biolegend, Ref. 902303	AB_2734670
	Rabbit anti-Foxg1, IgG	Foxg1	Synthetic peptide corresponding to Human FOXG1 aa 400 to the C-terminus (C terminal) conjugated to keyhole limpet haemocyanin	1:1,000	Abcam Antibodies, Ref. ab18259	AB_732415

**TABLE 2:** Secondary antibodies.

Type	Antibody	Dilution	Manufacturer and Reference
**Secondary antibodies**			
Biotinylated	Goat anti-chicken IgY (H+L), biotinylated	1:200	Vector, Ref. BA-9010
	Goat anti-rabbit IgG, biotinylated	1:200	Vector, Ref BA-1000
**Secondary antibodies**			
Fluorescent	Goat anti-chicken IgY (H+L), coupled to Alexa 488	1:500	Invitrogen, Ref. A-11039
	Donkey anti-rabbit IgG (H+L), coupled to Alexa 568	1:500	Invitrogen, Ref. A-10042
	Donkey anti-rabbit IgG (H+L), coupled to Cy5 Affinity Pure	1:500	Jackson ImmunoResearch, Ref. 711-175-152

### Immunofluorescence to Detect GFP

Free-floating sections were processed and incubated in the primary antibody as explained above. However, we employed a different secondary antiserum coupled to Alexa 488 (Table 2). After incubation and rinsing, the sections were mounted as explained above, and finally coverslipped using an antifading mounting medium (Vectashield Hardset Antifade mounting medium, Vector Laboratories Ltd.). Selected sections were processed for double immunofluorescence to detect GFP and Foxg1 (a transcription factor expressed in telencephalic cells). This procedure was used as previously described (Morales et al., 2021). To detect Foxg1, we used a rabbit anti-Foxg1 primary antibody, and a secondary antiserum coupled to Alexa 568 or Cy5 (Tables 1, 2).

### Primary Antibody

GFP and HA antibodies were validated on Western blots by the respective manufacturer, as explained next:

The chicken anti-GFP antibody recognized a single band of 25 KDa on Western blots of HEK293 transfected cell lysates, and a band at the same molecular weight on Western blots of transgenic mouse spinal cords (manufacturer’s data sheet). No staining was seen in “naïve” non-transfected cells. This antibody has been successfully used to detect enhanced GFP in Viaat-eGFP knockin transgenic mice (Aresh et al., 2017).
The rabbit anti-HA antibody was raised against the twelve amino acid sequence CYPYDVPDYASL derived from the human influenza hemagglutinin surface glycoprotein and recognizes the epitope YPYDVPDYA. On Western blots of cell lysates, it recognized a single band of 50 KDa (manufacturer’s data sheet).
The rabbit anti-Foxg1 antibody recognizes a band of 50 KDa on Western blots of mouse brain tissue lysate, which is blocked with the addition of the immunizing peptide (manufacturer’s data sheet). In mouse brain sections, it produces a staining pattern identical to that observed with *in situ* hybridization for Foxg1 (Dou et al., 1999; Crossley et al., 2001).

### Digital Photographs and Figures

Digital microphotographs from immunohistochemical sections were taken on a Leica microscope (DMR HC, Leica Microsystems GmbH) equipped with a Zeiss Axiovision Digital Camera (Carl Zeiss, Germany). Low magnification digital microphotographs were obtained with a stereoscopic microscope (2000C Zeiss) equipped with a Canon Digital Camera (EOS 450D, Canon Inc., Japan). Fluorescent material was analyzed with a confocal microscope (Olympus FV1000, Olympus Corporation, Japan). Selected digital images were adjusted for brightness and contrast with Adobe Photoshop 2021 (Adobe Inc., United States) or Corel PHOTO-PAINT 2012 or 2019 (Corel Corporation, Canada). Finally, the figures were mounted using CorelDraw 2012 or 2019 (Corel Corporation).

## Results

In the brain of the respective transgenic mice (either Otp-eGFP or Otp-Cre; Rpl22-HA), the anti-GFP and anti-HA antibodies produced staining patterns highly similar to those previously observed for Otp using *in situ* hybridization and immunohistochemistry in wild type mice (Acampora et al., 1999, 2000; García-Moreno et al., 2010; Morales-Delgado et al., 2011; Díaz et al., 2015; Morales et al., 2021). In particular, the cellular distribution observed with GFP and HA in the hypothalamus, preoptic area and amygdala was identical to that of Otp using *in situ* hybridization and immunohistochemistry. Moreover, colocalization of GFP and Otp was shown in the hypothalamus and amygdala of Otp-eGFP mice at E12.5 (Morales et al., 2021). However, while classical immunoreactivity technique for detecting Otp in wild-type animals only labels the cell nucleus, in the Otp-specific reporter transgenic mice, the anti-GFP and anti-HA antibodies stain cell somata and neurites (dendrites and axons in the Otp-eGFP and mostly proximal dendrites in the Otp-Cre; Rpl22-HA mice). The Otp-eGFP was used to study the Otp cells in more detail, as it produced high quality labeling from embryos to adults (including in animals of more than 300 days). The Otp-Cre; Rpl22-HA was used for confirmatory purposes regarding cell distribution, which was mostly comparable to that seen in the Otp-eGFP, although a few differences were also observed.

### Embryonic Stages

Otp cells were studied during embryonic development using Otp-eGFP reporter mice. In agreement with previous data on Otp expression, in the embryonic forebrain GFP was first observed in the supraopto-paraventricular domain (SPV) of the alar hypothalamus (Acampora et al., 2000; García-Moreno et al., 2010; Morales-Delgado et al., 2011; Díaz et al., 2015), as well as in the telencephalon-opto-hypothalamic domain (TOH), a newly identified division at the frontier between SPV and subpallium that coexpresses Otp and the telencephalic transcription factor Foxg1 (Morales et al., 2021). From E12.5, two groups of scattered cells appeared to split from the TOH: one group spread into the pallial amygdala; another group spread into the septopreoptic region and the cingulate cortex.

#### Pallial Amygdala and Adjacent Pallial Areas

**FIGURE 1:**
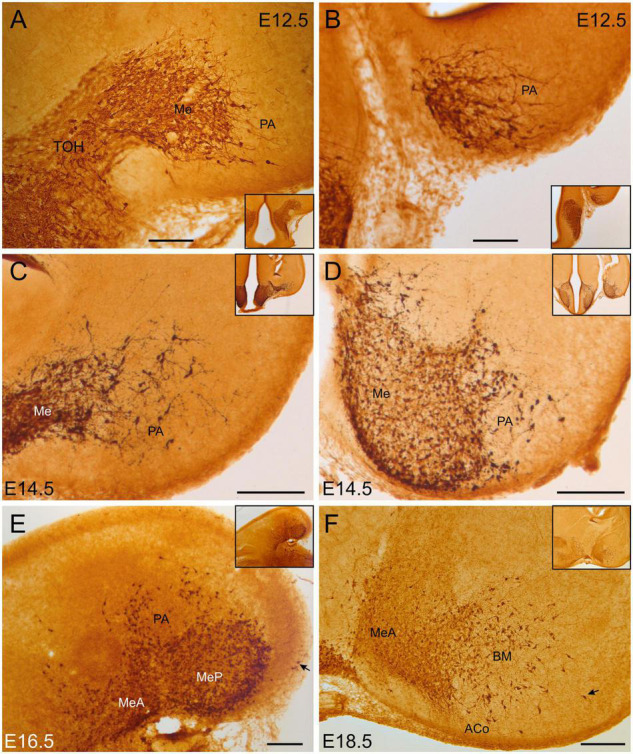
GFP immunoreactive cells in the embryonic amygdala. Images of brain sections of Otp-eGFP embryonic mice (E12.5 to E18.5), showing GFP immunoreactive cells in the developing amygdala. Note the continuity of pallial amygdala (PA) cells with those in the medial amygdala, which originate in the telencephalon-opto-hypothalamic domain (TOH). Panels **(A–D,F)** are frontal sections, while panel **(E)** is a horizontal section (for orientation, general views of the sections are shown in insets; in panel **(E)**, the posterior pole is to the right, and the medial side to the bottom). See text and abbreviation list for more details. Scale bar: panels **(A–D)** = 100 μm. Panels **(E,F)** = 200 μm.

From E12.5, some scattered GFP cells with migratory neuroblast morphology separated from the medial amygdala and reached the pallial amygdala (Figures 1A,B). At E14.5, scattered GFP cells were located in the primordia of the cortical amygdala and the basomedial amygdala (Figures 1C,D). From E16.5 onward, these cells were found within the anterior cortical amygdaloid area (ACo) and around the nucleus of the lateral olfactory tract (LOT), as well as in the basomedial amygdaloid nucleus (BM) (Figure 1E). Extremely few cells were seen in the vicinity of the piriform cortex. By E18.5, in addition to the previous groups, a few cells were seen in the posterior cortical amygdalar area (PCo), particularly in its medial part. Abundant varicose fibers were also observed in this posterior pole of the amygdala, which spread into the adjacent amygdalohippocampal and amygdalopiriform areas (Figure 1F).

#### Septopreoptic Region and Cingulate Cortex

**FIGURE 2:**
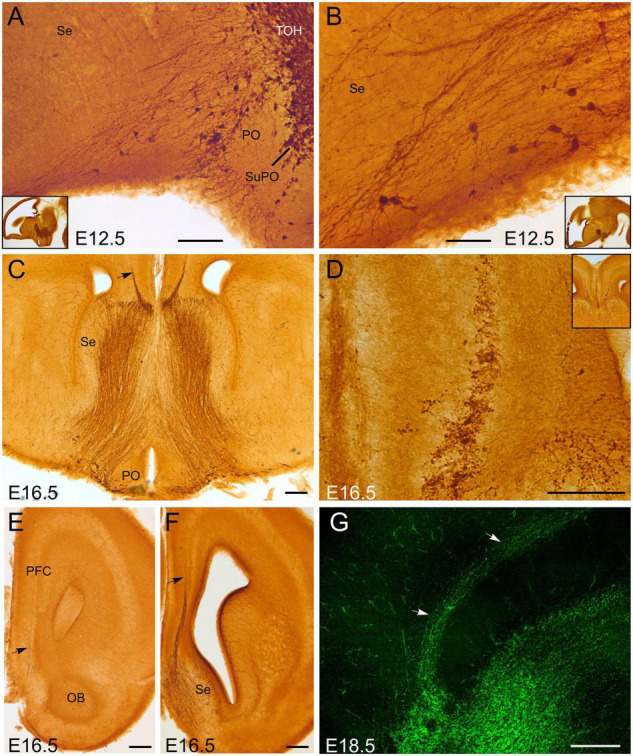
GFP immunoreactive cells and fibers in the embryonic septum and adjacent cortex. Images of brain sections of Otp-eGFP embryonic mice (E12.5 to E18.5), showing GFP immunoreactive cells and fibers in the preoptic area and septum **(A–C)**, and a fascicle of labeled fiber with intermingled cell bodies traversing the septo-cortical border [arrow in panel **(C)** and detail in panel **(D)**]. Labeled fibers and cells entering the cortex through this pathway are evident before the formation of corpus callosum and, later, at precallosal levels; from here they continue caudalwards as part of the cingulate bundle [arrows in panel **(G)**]. Panels **(A,B,G)** are sagittal sections; [panel **(G)** is fluorescent]; panel **(C)** is an oblique-horizontal section; [panel **(D)** is a detail of panel **(C)**]; panels **(E,F)** are frontal sections. Note the continuity of labeled fibers and cells of the septopreoptic region with those in the subpreoptic area (SuPO) of the TOH **(A)**. See text and abbreviation list for more details. Scale bar: panels **(A,D)** = 100 μm. panel **(B)** = 50 μm. panels **(C,E–G)** = 200 μm.

At E12.5, GFP cells of the subpreoptic area of TOH were in continuity with some cells found in the preoptic area, and a trail of scattered cells found in the septum (Figures 2A,B). Some of these cells showed a migratory neuroblast morphology. This GFP cell trail along the septopreoptic region was accompanied by some GFP fibers, which appeared to originate (at least partially) in the GFP cell groups of TOH and possibly SPV (including those of the subpreoptic area, extended amygdala and paraventricular hypothalamus). At E16.5, the projection through the septopreoptic region dramatically increased (Figure 2C), and cells along the trail became more abundant, with a decreasing gradient from preoptic area to septum. The fibers provided a strong innervation in this whole region. In addition, a small group of fibers left the septum and entered the pallium at pre-callosal levels, and appeared to form a distinct GFP-labeled fascicle of the cingulate bundle (Figures 2C–F), which was more evident in sagittal sections at E18.5 (Figure 2G). This fascicle ran longitudinally from frontal to retrosplenial levels of the cortex, just above corpus callosum. Small cell bodies appeared intermingled among the fibers of the cingulate bundle at E16.5 and E18.5. Varicose fibers were also observed adjacent to the bundle, in the vicinity of the subplate and cortical white layer, as well as in the marginal layer of the cingulate cortex.

### Postnatal Stages and Adults

**FIGURE 3:**
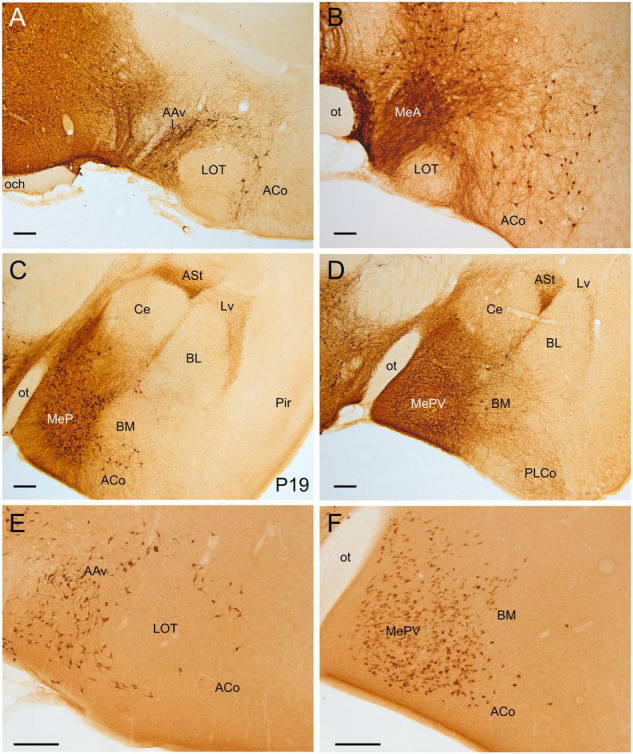
GFP and HA immunoreactivity in the early postnatal and adult amygdala. Images of frontal brain sections of Otp-eGFP mice **(A–D)** and Otp-Cre; Rpl22-HA mice **(E,F)**, showing GFP or HA immunoreactive cells in the postnatal [**(C)**, P19] and adult **(A,B,D–F)** amygdala. Note the highly similar distribution of cells in the medial and pallial amygdala in both transgenic mice. In the Otp-eGFP mice, the neuropil also shows immunoreactivity, allowing analysis of Otp-specific networks. See text and abbreviation list for more details. Scale bar: panels **(A–F)** = 200 μm.

**FIGURE 4:**
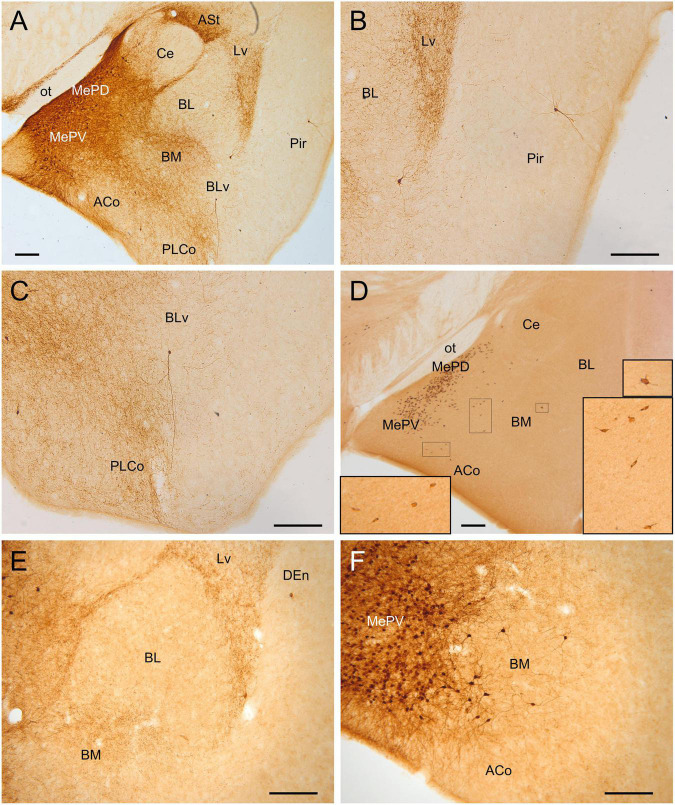
GFP and HA immunoreactivity in the adult amygdala. Images of frontal brain sections of adult Otp-eGFP mice **(A–C,E,F)** and Otp-Cre; Rpl22-HA mice **(D)**, showing GFP or HA immunoreactive cells in the amygdala. In the Otp-eGFP mice, the neuropil also shows immunoreactivity, allowing analysis of Otp-specific networks. Note the highly similar distribution of cells in the medial and pallial amygdala in both transgenic mice. In the pallial amygdala, some cells are seen in the anterior cortical amygdalar area (ACo) and the basomedial nucleus (BM), but a few cells are also seen in the ventrolateral part of the lateral nucleus (Lv) and the ventral part of the basolateral nucleus (BLv). In addition, a few cells are visible in the endopiriform nucleus and piriform cortex. See text and abbreviation list for more details. Scale bar: panels **(A–F)** = 200 μm.

**FIGURE 5:**
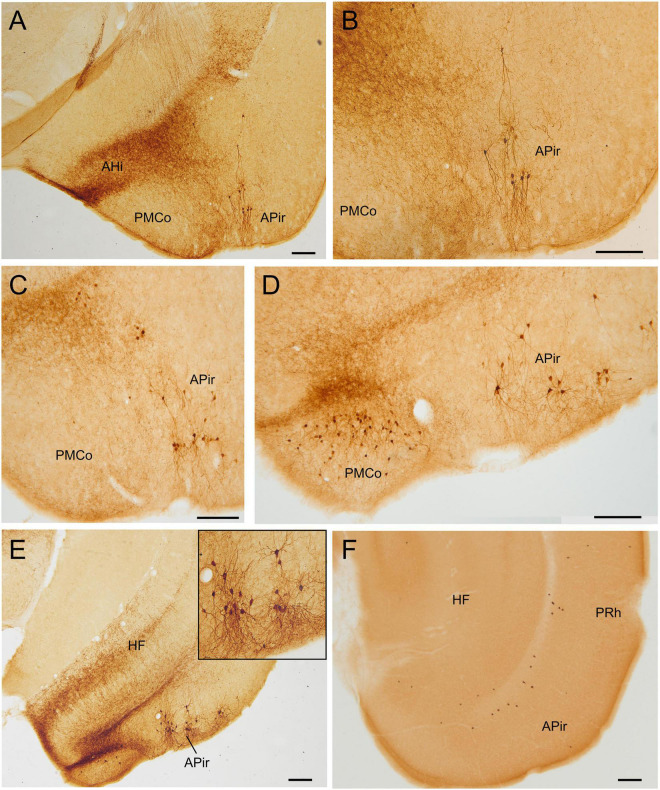
GFP and HA immunoreactivity at posterior levels of the adult amygdala. Images of frontal brain sections of adult Otp-eGFP mice **(A–E)** and Otp-Cre; Rpl22-HA mice **(F)**, showing GFP or HA immunoreactive cells in the posterior cortical amygdalar area (in particular PMCo) and adjacent amygdalo-piriform transition area (APir). Dense neuropil is also observed in the amygdalo-hippocampal area (AHi) and adjacent hippocampus. In the Otp-Cre; Rpl22-HA, labeled cells are also observed in similar positions (such as APir), but most of them occupy a deeper stratum than in the Otp-eGFP. See text and abbreviation list for more details. Scale bar: panels **(A–F)** = 200 μm.

**FIGURE 6:**
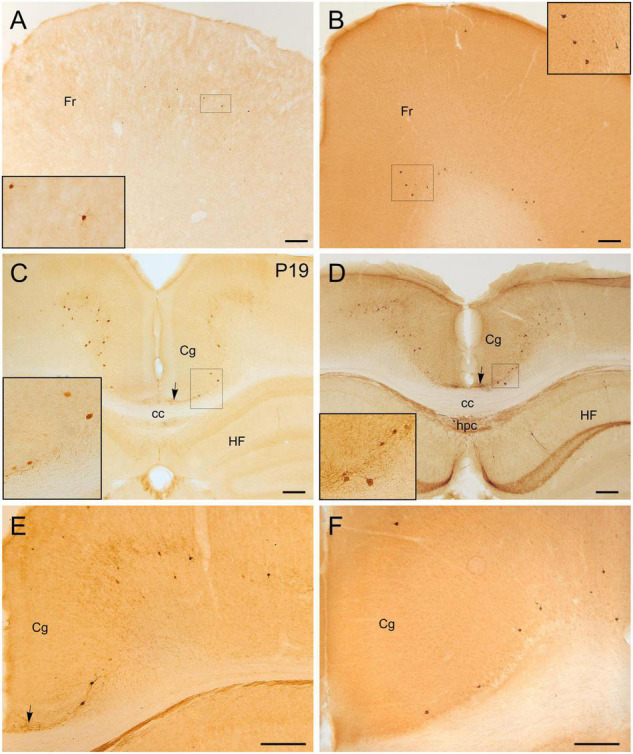
GFP and HA immunoreactivity in the cerebral cortex, including the cingulate cortex. Images of frontal brain sections of adult Otp-eGFP mice **(A,C–E)** and Otp-Cre; Rpl22-HA mice **(B,F)**, showing GFP or HA immunoreactive cells in the mesocortex and neocortex, including frontal **(A,B)** and cingulate areas **(C–F)** (details of cells in insets). A fascicle of labeled fibers is seen in the cingulate bundle [arrows in panels **(C–E)**] in the Otp-eGFP mice, and a group of cells appear in the vicinity of it in the cingular cortex [insets in panels **(C,D)**]. In the Otp-Cre; Rpl22-HA, labeled cells are observed in similar positions of the mesocortex and neocortex to those in the Otp-eGFP, but many of them occupy a deeper stratum than those in the Otp-eGFP. See text and abbreviation list for more details. Scale bar: panels **(A–F)** = 200 μm.

**FIGURE 7:**
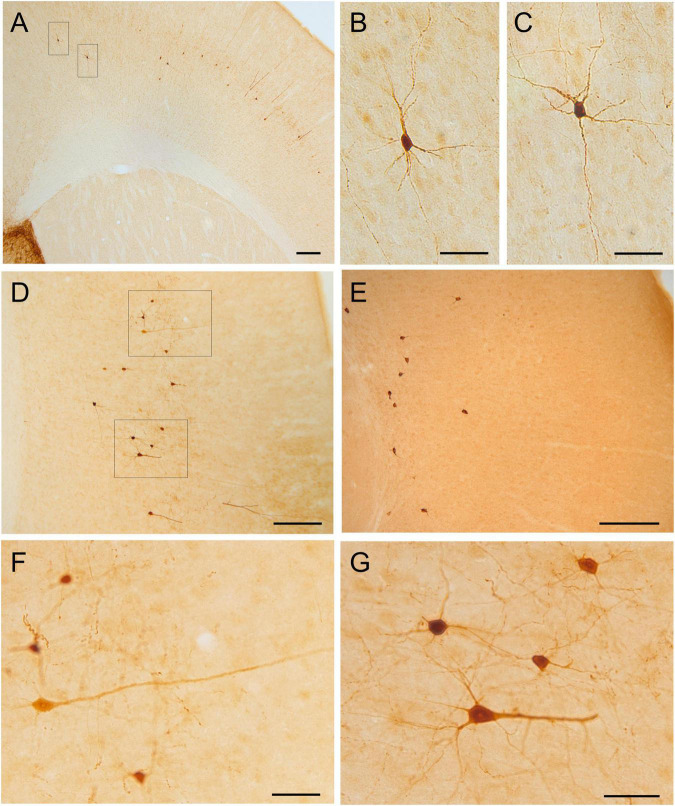
GFP and HA immunoreactivity in the neocortex. Images of frontal brain sections of adult Otp-eGFP mice **(A–D,F,G)** and Otp-Cre; Rpl22-HA mice **(E)**, showing GFP or HA immunoreactive cells in the mesocortex and neocortex, from medial to lateral levels. Panel **(A)** shows the mesocortex and the adjacent medial part of the neocortex, while panels **(D,E)** show lateral parts of the neocortex (the images were rotated, so that the pial surface is to the right). Details of the cells are displayed in panels **(B,C,F,G)**, showing their highly heterogeneous morphologies, ranging from large pyramidal like to small multipolar or stellate types. In the Otp-Cre; Rpl22-HA, labeled cells are observed in similar positions of the cortex to those in the Otp-eGFP, but many of them occupy a deeper stratum than those in the Otp-eGFP. See text and abbreviation list for more details. Scale bar: panels **(A,D,E)** = 200 μm. Panels **(B,C,F,G)** = 50 μm.

**FIGURE 8:**
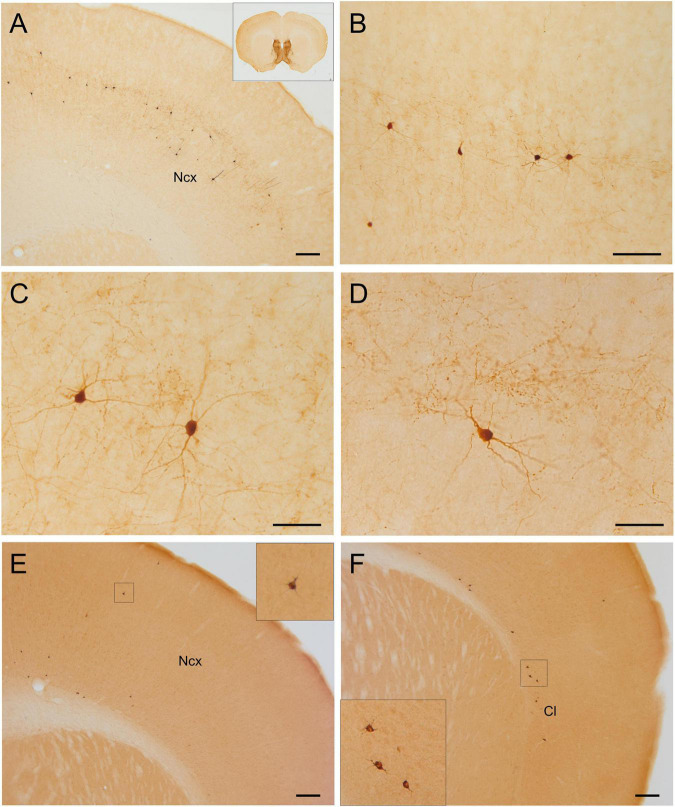
GFP and HA immunoreactivity in the neocortex. Images of frontal brain sections of adult Otp-eGFP mice **(A–D)** and Otp-Cre; Rpl22-HA mice **(E,F)**, showing GFP or HA immunoreactive cells in the neocortex **(A–F)** and claustro-insular region **(F)**. Panels **(B,C,D)** display details of neocortical cells from the section shown in panel **(A)**. Scale bar: panels **(A,E,F)** = 200 μm. Panel **(B)** = 100 μm. Panels **(C,D)** = 50 μm.

**FIGURE 9:**
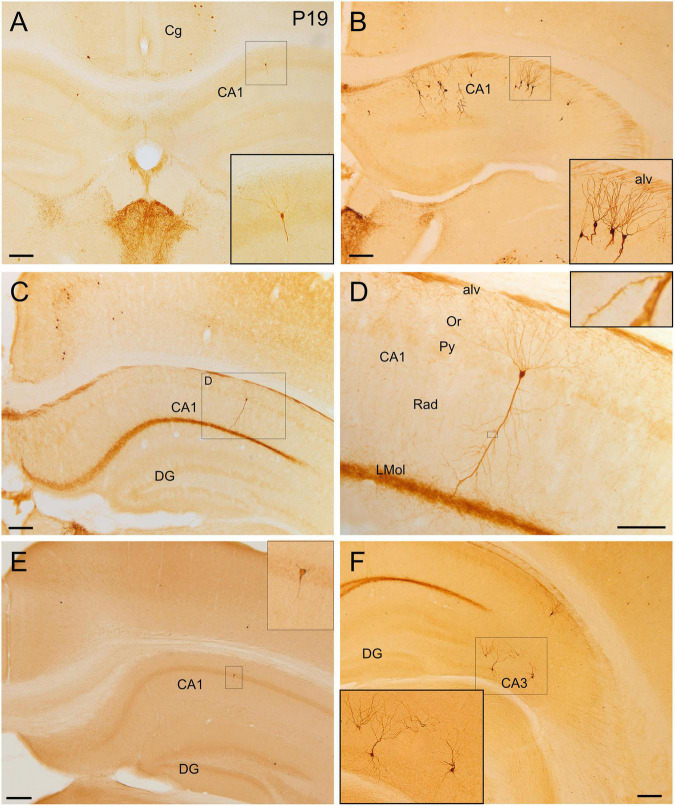
GFP and HA immunoreactivity in the postnatal and adult hippocampal formation. Images of frontal brain sections of postnatal **(A)** and adult **(B–D,F)** Otp-eGFP mice and adult Otp-Cre; Rpl22-HA mice **(E)**, showing GFP or HA immunoreactive cells in the hippocampal formation. In particular, the images show cells in different CA subfields (details in insets). In the Otp-Cre; Rpl22-HA, labeled cells are observed in similar positions of the hippocampal formation to those in the Otp-eGFP. In the CA fields, cells are found in the pyramidal cell layer and show a pyramidal-like morphology, with spiny dendrites [inset in panel **(D)**]. Basal and apical dendrites traverse the strata and are in contact with labeled axons of the alveus (alv) and stratum lacunosum moleculare (LMol). See text and abbreviation list for more details. Scale bar: panels **(A–C,E,F)** = 200 μm. Panel **(D)** = 100 μm.

**FIGURE 10:**
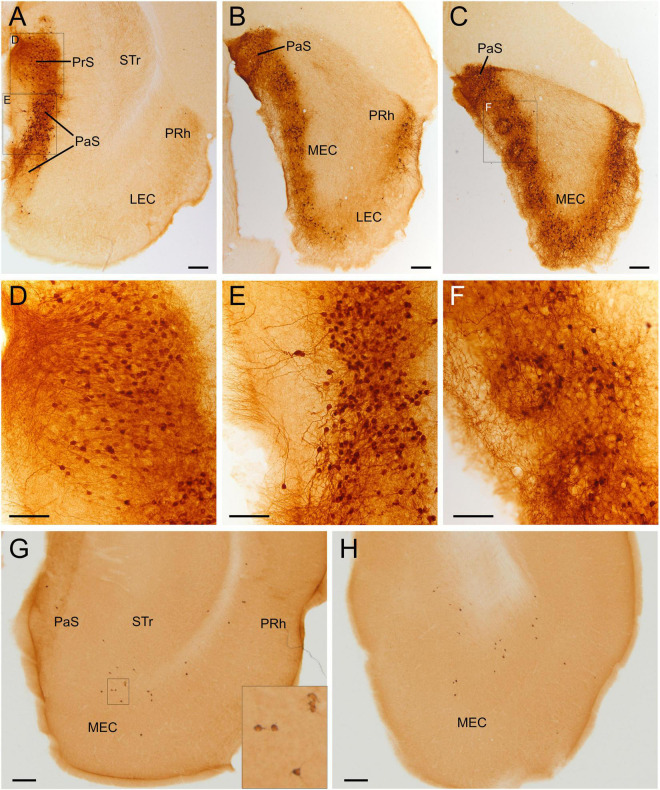
GFP and HA immunoreactivity in the adult parahippocampal lobe. Images of frontal brain sections of adult Otp-eGFP mice **(A–F)** and adult Otp-Cre; Rpl22-HA mice **(G,H)**, showing GFP immunoreactive cells and neuropil (neuropil only seen in the Otp-eGFP) in several areas of the parahippocampal formation, including the presubiculum [**(A)**, detail in panel **(D)**], the parasubiculum [**(A–C)**, detail from area squared in panel **(A)** in panel **(E)**] and the caudomedial entorhinal cortex [**(B,C)**, detail from area squared in panel **(C)** in panel **(F)**]. Note the location of the labeled cells around the patches or islands of the medial entorhinal cortex. In the Otp-Cre; Rpl22-HA, labeled cells are observed in similar positions of the parahippocampal lobe to those in the Otp-eGFP, but in the first mouse line most cells occupy a deeper stratum **(G,H)**. See text and abbreviation list for more details. Scale bar: panels **(A–C,G,H)** = 200 μm. Panels **(D–F)** = 100 μm.

The first postnatal age we studied using the Otp-eGFP mice was P6. At this age and later (P12, P19, adults), GFP cells continued to be observed in specific subdivisions of the pallial amygdala (Figures 3–5), as well as along and adjacent to the cingulate bundle (bundle pointed with an arrow in Figures 6C–E). However, neuron maturity and neuropil density gradually increase with age. Moreover, from P6 we started to observe new populations of GFP cells inside the cingulate mesocortex (Figure 6) as well as in several areas of the neocortex (Figures 7, 8). From P60, additional populations were observed in the claustro-insular region, the hippocampal formation (Figures 8, 9) and, more caudally, in the caudalmost aspect of the perirhinal cortex (Figure 10). In adult animals, striking groups of GFP cells were observed in different parts of the parahippocampal lobe, including the post-, pre- and para-subiculum, and the caudomedial part of the entorhinal cortex (Figures 10A–F). The presence of Otp cells in most of these different areas of the pallium was confirmed in the adult Otp-Cre; Rpl22-HA mice (Figures 3E,F, 4D, 5F, 6B,F, 7E, 8E,F, 9E, 10G,H). In both mouse lines, the latest labeled cells observed were those of the parahippocampal lobe (seen in adult animals, but not before), while other Otp-specific pallial cells are seen from early postnatal ages if not earlier. However, while the distribution of Otp-specific labeled cells in the postnatal/adult pallium was highly similar in both transgenic mice, in the neocortex and parahippocampal lobe of the Otp-Cre; Rpl22-HA mice, most labeled cells were located in deeper layers, when compared to their location in the Otp-eGFP mice. Below we described the Otp cells in the postnatal pallium based on observations in the Otp-eGFP.

#### Pallial Amygdala and Adjacent Olfactory Area

Postnatally, we continued to observe GFP cells in the basomedial nucleus of the amygdala, as well as in the anterior and posterior cortical amygdalar areas (Figures 3–5). Regarding the latter, cells were visible in the posterior aspect of the posteriomedial cortical amygdalar area and the adjacent amygdalo-hippocampal area (PMCo, AHi, Figures 5D,E). Many GFP cells of the basomedial nucleus were multipolar, with a large cell body and long dendrites or processes. Often, these cell processes extended into the cortical amygdalar areas, into other parts of the basomedial nucleus and/or into the medial amygdala. In addition, few cells were also present in the ventral subnuclei of the lateral and basolateral amygdala (Figures 4A–C,E). All subdivisions of the pallial amygdala containing GFP cell bodies also contained a moderate to dense GFP immunoreactive neuropile, and all together appeared to form a GFP positive network, in continuity with that of the medial amygdala (Figures 3B–D, 4F). Extremely few GFP cells were also observed in the endopiriform nuclei, as well as layers 1 and 3 of the piriform cortex, scattered from rostral to caudal levels (Figures 4B,E). Those in layer 1 were tangentially oriented, and were mainly seen at rostral levels. Those in layer 3 were radially oriented and were seen at intermediate and caudal levels of the piriform cortex. Moreover, from P19 a distinct cell group was also observed in the amygdalopiriform transition area, at caudal levels. In adult animals, cells of this group showed a patchy organization, with sets of radially arranged cells surrounding GFP-poor areas (Figures 5C–E). In addition, a group of GFP cells was found in the caudalmost part of the perirhinal cortex (Figure 10B).

#### Cingulate Mesocortex and Bundle

**FIGURE 11:**
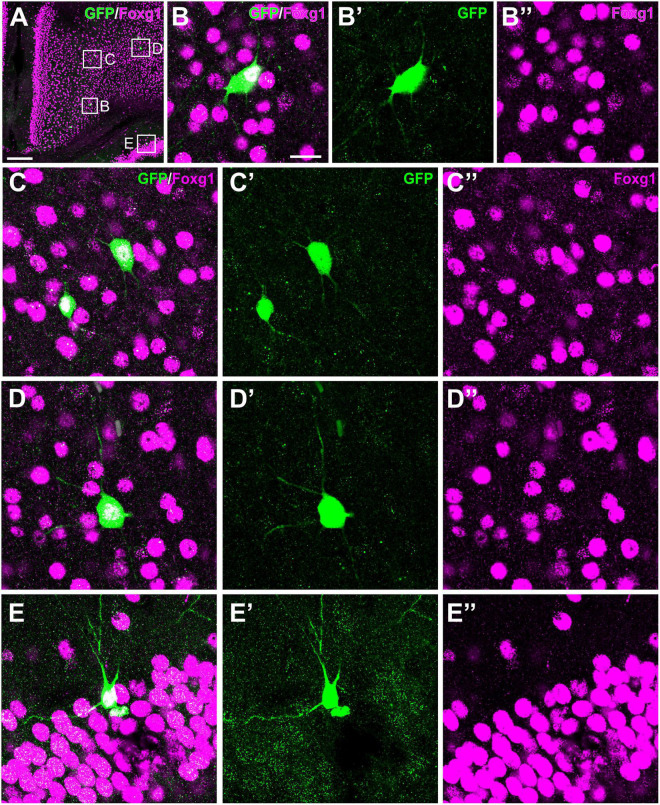
Colocalization of GFP and Foxg1 in the mesocortex, neocortex and hippocampus. Images of frontal brain sections of P19 Otp-eGFP mice, processed for double immunofluorescence to detect GFP (green) and Foxg1 (magenta). Panel **(A)** shows the cingulate mesocortex and adjacent neocortex, and details of double labeled cells are shown in panels **(B–B′′,C–C′′,D–D′′)**. Panels **(E–E′′)** shows a double labeled cell in CA1. Scale bar: panel **(A)** = 200 μm. Panel **(B)** = 20 μm [it applies to panels **(B–E′′)**].

Postnatally, a few GFP cells were observed in the cingulate cortex, some adjacent to the cingulate bundle and others at various distances (Figure 6). Most of these cells were located in deep layers and showed a roughly round or polygonal small soma with several dendrites. A few also displayed a thin process (perhaps the axon) extended into the cingulate bundle (Figure 6C, detail in inset; arrow points to the cingulate bundle). Initially (P6, P12), these GFP cells were mostly located at frontal levels of the cingulate cortex, but they were progressively found more caudally. At P19 and later, they were also observed in the retrosplenial cortex. These cells were double labeled with Foxg1 (Figure 11). Similar cells were also identified in comparable locations in the Otp-Cre; Rpl22-HA mouse (Figures 6B,F). Moreover, GFP and HA cells were found in similar locations in both hemispheres, displaying a roughly symmetrical distribution.

#### Neocortex

Postnatally, a few GFP cells started to be observed in the neocortex, including frontal, parietal and occipital areas (Figures 6–8). These included different types, which located in different layers: (1) A few were bipolar cells, located in a superficial layer (layers 2 or 3), and showed a tangential organization. (2) Other cells were found in layers 3–5; these included two morphological types: most were multipolar with round or polygonal soma, which were equidistant from one another, and seemed to be interconnected (these were morphologically similar to those in the cingulate cortex mentioned above); other GFP cells had a pyramidal-like morphology (Figure 7D, and details in Figures 7F,G). (3) Other cells were located in the deepest layer (layer 6), adjacent to the white matter, and were often oriented parallel to it (some of these might be subplate cells). Most non-pyramidal cells had a small soma with aspiny or sparsely spiny beaded dendrites (Figures 7G, 8B–D), but the pyramidal-like cells were larger and had spiny dendrites (Figures 7F,G). Similar cells were also observed in comparable areas of the neocortex of the adult Otp-Cre; Rpl22-HA mouse, but they were more abundant in the deepest layer, adjacent to the white matter (Figures 7E, 8E,F). As above, GFP and HA cells displayed a roughly symmetrical distribution in the neocortex.

#### Hippocampal Formation

Few GFP cells were also observed in different areas of the hippocampal formation at least from P19. At rostral hippocampal levels, a few of these cells were seen in the dentate gyrus, CA3, CA2 and CA1, and subiculum (Figure 9). These cells were a bit more abundant and showed stronger immunoreaction in adults. In the dentate gyrus, extremely few cells were found in the polymorphic cell layer and the granular cell layer. In the CA field, GFP cells were located in the pyramidal cell layer and displayed a pyramidal-like morphology, with striking spiny dendrites spanning all layers (Figures 9A–D; detail in Figure 9D and its inset). Basal dendrites extended through the *stratum oriens* (which contained varicose axons) and reached the alveus, and their tips were in contact with GFP positive axons running in the alveus, in continuity with the hippocampal commissure. The apical dendrite extended and branched through the *stratum radiatum* (where associational connections between CA fields are found) and reached the *stratum lacunosum moleculare* (the terminal zone of the perforant pathway, with the projection from the entorhinal cortex; Witter and Amaral, 2004), which displayed a densely labeled neuropil. GFP cells of the hippocampal formation coexpressed Foxg1 (Figures 11E–E′′). The distribution of labeled cells in the hippocampal formation observed in the Otp-eGFP mouse was similar to than seen in the Otp-Cre; Rpl22-HA mouse (Figure 9E).

#### Parahippocampal Areas

A striking, large group of GFP cells was observed in the parahippocampal complex of adult animals, in particular in the pre- and para-subiculum, as well as in the caudomedial entorhinal cortex (Figure 10). A few cells were also observed in the postsubiculum. In the presubiculum, cells were located in superficial layers 2/3, showed bipolar or small pyramidal-like morphology, and were oriented perpendicular to the surface, with apical processes radially extending into superficial layer 1 (Figure 10A, detail in Figure 10D). In the parasubiculum, cells were mostly located in layer 2, and had a round morphology with apical dendrites oriented toward the surface (Figures 10A–C; a detail from Figure 10A is shown in Figure 10E). Both, pre- and para-subiculum contained a densely immunoreactive neuropil, which extended into the superficial layer 1 (Figures 10A–E). In the caudomedial entorhinal cortex, GFP cells displayed a ring-like arrangement around GFP cell poor areas or islands (see detail in Figure 10F). Most GFP cells of this caudal pole of the pallium were located in layer 2 (around GFP cell free islands) and adjacent layer 3. Many of the cells displayed round somata and long dendrites extending into superficial layer 1, but somata around islands also displayed processes extending into the island center (Figure 10F). All GFP cells of the parahippocampal areas were immersed in a densely immunoreactive neuropil, and at least those of the caudomedial entorhinal cortex appeared to be the origin of abundant GFP axons seen in the deep white matter/subiculum transition zone, which continued rostrally toward the alveus of the hippocampal formation. Parahippocampal areas also contained some labeled cells in the Otp-Cre; Rpl22-HA mouse, but they were fewer and most of them appeared to occupy a deeper position (Figures 10G,H).

## Discussion

### General Findings

Using two Otp-specific reporter lines of transgenic mice, our results provide new evidence for the presence of different populations of Otp cells in various areas of the pallium, including the pallial amygdala, the neocortex, the piriform cortex, the claustrum and endopiriform nuclei, the hippocampal formation and the parahippocampal lobe. The presence of Otp cells in the pallial amygdala was previously reported (García-Moreno et al., 2010; Morales et al., 2021), but the description of Otp cells in other pallial areas such as the neocortex, hippocampal formation and parahippocampal lobe is new. Nevertheless, our data are consistent with results on scarce, but strong mRNA expression of Otp in the meso- and neocortex (specially the cingulate and frontal cortex) and entorhinal cortex of pig, mouse and human (see text footnote 3; data from RNA-seq). The fact that these cells are found in the cortical mantle postnatally explains why they were not seen in previous studies on expression of Otp in mouse brain sections, since most of these were done during embryonic development. This might also be due to the scarcity of these cells (except those of the parahippocampal lobe, but these appeared very late), which makes it difficult to see them in thin hybridized sections, like those of the Allen Mouse Brain Atlas (nevertheless, in enlarged images from brains at P56 of the Allen Atlas is possible to observe a few cells in different areas of the pallium). Transgenic mice carrying BAC vectors, like the Otp-eGFP employed here, show increased sensitivity of the reporter gene and allow detection of gene expression in sites that are not evident using *in situ* hybridization or immunohistochemistry (Gong et al., 2003). This type of methodology, together with the increased stability of the enhanced GFP, allow identification of expressing cells not seen with conventional methods, providing a more faithful reproduction of endogenous expression, in addition to high precision details on cell dendrites and axons (Schmidt et al., 2013). Taking advantage of the Otp-eGFP transgenic mouse, we could identify new cell populations in the mouse pallium. These cells are consistently found in two lines of Otp-specific reporter transgenic mice, as well as across animals (for each age and mouse line, we analyzed many different cases), giving support to our data. Future studies, employing highly sensitive methodologies, will need to be done to further corroborate these data. Our results provide evidence for the presence of several types or subtypes of Otp cells in the pallium, and raise questions on their developmental origin, molecular features, degree of evolutionary conservation, connections and functions. Intriguingly, in the mesocortex, neocortex and parahippocampal lobe of the Otp-Cre; Rpl22-HA mice, labeled cells showed a trend to locate in a deeper stratum, compared to cells in the same areas in the Otp-eGFP mice. More research needs to be done to investigate the reason for this difference, but it might be due to a problem in migration of some populations of cortical/pallial cells in the Otp-Cre; Rpl22-HA mice.

### Multiple Subtypes of Otp Cells in the Pallium

One important aspect to consider is that Otp cells of the pallium are non-uniform and include different types depending on their location and morphology, the moment when they start to express Otp (embryonic vs. postnatal onset), their neuronal or non-neuronal category, their embryonic origin (local/pallial vs. external/non pallial), and other aspects such as their putative glutamatergic or GABAergic nature, or if they are involved in long-range or local connections.

#### Embryonic Onset of Otp and Cellular Origin

The early onset of Otp expression suggests a role of this transcription factor in development. Previous studies have shown that it is involved in differentiation of dopaminergic and neuroendocrine cells in the hypothalamus and other brain areas (Acampora et al., 1999, 2000; Wang and Lufkin, 2000; Ryu et al., 2007; Biran et al., 2015), and in migration of cells to the amygdala (García-Moreno et al., 2010). Based on our observations, it appears that pallial cells with embryonic onset of Otp expression have an extrinsic origin, and Otp might be playing a role in the migration of these cells to their final destination. We have two different cases: cells of the pallial amygdala and cells intermingled and adjacent to the cingulate bundle.

*Otp cells of the pallial amygdala:* A previous experimental study showed that, during embryonic development, Otp cells of the preoptic area, medial bed nucleus of the stria terminalis (BSTM) and medial amygdala originate in the SPV hypothalamic domain (García-Moreno et al., 2010), and we showed that the vast majority of these cells specifically originate in the TOH domain, at the frontier between telencephalon and hypothalamus, and coexpress Otp and Foxg1 (Morales et al., 2021). It is likely that Otp cells of the pallial amygdala also originate in TOH, since they are observed from early embryonic stages in clear continuity with those of the medial amygdala. From early stages, we observed labeled processes extended between the medial extended amygdala and areas of the pallial amygdala containing Otp cells. This suggests that Otp cells born in the TOH domain establish connections between them at an early stage, and remain connected along the migratory route to the medial and pallial amygdala. If confirmed, this would provide an explanatory mechanism for the formation of a functional network between Otp cells of the pallial amygdala, extended amygdala, and dorsal parts of the paraventricular region (all derived from TOH; Morales et al., 2021).

*Otp cells of the cingulate bundle and adjacent cortex:* Our results showed GFP labeled fibers and migratory neuroblasts traversing the septum from E12.5, and at E16.5 a subset was already observed entering the pallium at precallosal levels. Labeled fibers formed a distinct fascicle of Otp axons within the cingulate bundle, and were accompanied by intermingled labeled cells. Otp may be playing a role in the development of this fascicle and the migration of the cells. Moreover, the location of cells intermingled between the axons suggests that some of these cells may play a role as axonal guideposts during fascicle development and/or might be using the fibers as guidance for migration, as shown elsewhere (for example, Tinterri et al., 2018). The labeled fibers of the cingulate bundle might primarily represent the projection from Otp cells located in subpallial and TOH telencephalon (preoptic and subpreoptic area, extended amygdala) and adjacent hypothalamus, but one intriguing question is where are the cells intermingled with the fibers coming from. Also, do they contribute to form a subset of Otp cells observed postnatally in the cingulate cortex? Regarding the first question, at least some of them appear to coexpress GFP and Foxg1, suggesting a TOH origin (but see more below). With respect to the second question, they might produce some Otp cells of the cingulate cortex adjacent to the cingulate bundle. Some of these neurons were seen to extend a process toward it. This subset of cells of the cingulate cortex might have an external origin and might have invaded the cortex following the fibers. However, other mesocortical and neocortical Otp cells might have a local origin inside the pallium, and experimental cell migration assays are needed to discern between both possibilities. All of these cortical cells coexpress GFP and Foxg1, although this cannot help distinguishing between an external origin in TOH or inside the pallium.

#### Late Onset of Otp in Cells of the Neocortex, Hippocampus and Parahippocampal Lobe

As noted above, subsets of Otp cells are seen in different areas of the mesocortex/neocortex, hippocampus and parahippocampal lobe postnatally. It is likely that most of these cells are produced locally, although we cannot discard the possibility that at least some of them are produced from cells that migrated to the pallium during embryonic development and remained as quiescent progenitors in the white matter. The onset of Otp expression in neocortical cells depends on the area: based on GFP and HA expression in the reporter mice, those in the neocortex show Otp expression at least from P6 onward, those in the hippocampal formation from P19 onward, and those in the parahippocampal lobe in adults. The role of Otp in these mature cells is unclear and awaits further investigation. As discussed above, this transcription factor plays a very important role during development of specific subsets of neurons of the hypothalamus, amygdala and other brain areas (reviewed by Biran et al., 2015). However, in some of these areas, including the hypothalamus and amygdala, expression of Otp is maintained throughout ontogenesis. In the adult hypothalamus, Otp may play a role in regulating the expression of genes relevant for the physiological functions in which this brain region is involved, such as homeostasis maintenance (Biran et al., 2015). Similarly, in the adult brain, Otp may play various roles in modulating neocortical, hippocampal and parahippocampal functions. The specific role will depend on the interactions of Otp with other transcription factors and molecules present in the cell. Thus, studies on single cell transcriptome of different Otp pallial cells will surely help to clarify the adult functions of this transcription factor.

### Cell Types and Neurotransmitters

Based on their morphology, many of the GFP labeled cells of the pallium seem to be neurons, but we also observed non-neuronal typologies. This agrees with results of Otp expressing cells in the cerebral cortex of the Human Protein Atlas, where they found that part of the cells are neurons, while other cells are glial or endothelial cells. In a previous study, we also found Otp (but not GFP) in the endothelium of developing blood vessels of the hypothalamus and telencephalon in the Otp-eGFP reporter mouse (Morales et al., 2021). In the present study we found GFP and HA immunoreactive small cell bodies in the white matter, such as those intermingled in the cingulate bundle, in the white matter subjacent to the neocortex or related to the hippocampal fiber association systems. Some of these may be oligodendrocytes or other type of glial cells, but others may be undifferentiated neuronal precursors.

Regarding the neurons, the majority of those found in the telencephalon, including most of those of the pallium, are likely glutamatergic, as shown for the extended amygdala and paraventricular hypothalamic nucleus using multiple labeling (García-Moreno et al., 2010; Morales et al., 2021). This is likely independent of their origin in TOH or inside the pallium, since both divisions typically produce glutamatergic neurons (Hevner et al., 2001; Osório et al., 2010; Morales et al., 2021). However, we should note that there is a very small percentage of cells in the subpallial telencephalon that coexpress Otp and either the transcription factor Dbx1 or Foxp2; these two transcription factors define two different subtypes of GABAergic neurons derived from the preoptic embryonic domain (Lischinsky et al., 2017; see their supplementary figure 1). Thus, the possibility exists that a small percentage of the Otp cells of the pallium are GABAergic. In fact, some of those found in the neocortex resemble subtypes of GABAergic neurons of the neocortex.

### Networking

How are the different Otp neurons of the pallium connected? Are they projection neurons or local circuit neurons? In case they are projection neurons, could they be involved in pallio-pallial associational connections and/or in descending projections? We will analyze these questions separately for the pallial amygdala, the mesocortex/neocortex and the hippocampal complex. Before starting, it is important to clarify concepts to avoid classical views on GABAergic neurons of the pallium as local circuit cells and glutamatergic neurons as associational or long-range descending neurons because this may lead to erroneous conclusions. Different studies have revealed GABAergic neurons of the neocortex, including subtypes of VIP or parvalbumin neurons, with long-range projections to the striatum, amygdala and/or thalamus (for example, Lee et al., 2014; Bertero et al., 2021). Similarly, some glutamatergic cells of the neocortex may preferentially have local or within-column connections, such as those of layer 4 (for example, Scala et al., 2019).

#### Pallial Amygdala

According to our results, Otp neurons of the pallial amygdala represent a small subpopulation, but they are large and multipolar, and seem to belong to an Otp network extended across the cortical amygdalar areas, basomedial amygdalar nucleus and medial amygdala (see explanations above). Thus, these Otp neurons seem to project preferentially across this network, but the exact connections and function of this network needs to be investigated. Based on the known general connections of the pallial amygdala areas/nuclei that contain Otp cells (basomedial amygdala nucleus, Petrovich et al., 1996; PMCo, Kemppainen et al., 2002), they may modulate the activity of the medial amygdala, medial bed nucleus of the stria terminalis, the preoptic and subpreoptic areas and the hypothalamus (among other areas), and might play a role in regulating some aspects of social behavior and cognition. Another important question is to know how this Otp-specific network interacts with other networks of the amygdala, such as those regulating sexual or agonistic behaviors (Choi et al., 2005; Lischinsky et al., 2017). Cells of this network may also project to the central amygdala, which medial part is innervated by Otp fibers (present results, in Figures 3D, 4A), and thus could modulate the function of the central amygdala in the control of fear or anxiety responses (Phelps and LeDoux, 2005). Moreover, Otp cells of PMCo might be involved in projections to the parasubiculum (Kemppainen et al., 2002), and may play a relevant role in regulating parahippocampal/hippocampal functions.

#### Mesocortex and Neocortex

Our results show the presence of different types of Otp-related cells in the mesocortex and neocortex, some of which are found at embryonic stages, while other cells are found postnatally. Immature Otp cells are seen intermingled with the fibers of the developing cingulate bundle from middle embryonic stages, which suggest that some of these cells may play a role as axonal guideposts during fascicle development and/or might be using the fibers as guidance for migration (Tinterri et al., 2018). Postnatally, small subpopulations of cells are found in the mesocortex and neocortex, and are more abundant at frontal levels of the cingulate cortex, neighboring the cingulate bundle. Based on data from the Otp-eGFP mouse, Otp cells of the cingulate cortex may receive input and/or project through the cingulate bundle. This is a non-unitary bundle that interconnects medial parts of the mesocortex (from orbitofrontal and anterior cingulate to retrosplenial areas), hippocampal and parahipoccampal areas, but also carries fibers linking these areas with subcortical regions, such as the amygdala, diagonal band nuclei, hypothalamus, thalamus, locus coeruleus and raphe nuclei (Bubb et al., 2018). In humans, it has been involved in attention, motivation, emotion, pain, and episodic memory, but its complex nature makes it necessary to dissect its different components to better understand its implication in so many functions and the consequences of its dysfunction (Bubb et al., 2018). The Otp-specific reporter mice used in this study offer the opportunity to dissect and study one of its multiple components, consisting of Otp axonal projections.

In addition to the cells of the cingulate cortex (from anterior cingulate to retrosplenial areas) that might receive input and/or project through the cingulate bundle, we also observed small subpopulations of Otp cells in the neocortex. Some of them, located in layers 3–5, showed an equidistant distribution and were immersed in a network of Otp varicose fibers, suggesting that they were interconnected. If confirmed, these cells might play a role in synchronization of cortical columns. Again, the Otp-specific reporter mice used in this study offer the opportunity to study the connections and functions of these specific cells.

#### Hippocampal Complex

One of the most striking findings of this study is the presence of a large amount of Otp cells in the parahippocampal lobe, including the pre- and parasubiculum and the caudomedial entorhinal cortex of adult animals. As noted above, our data agree with previous findings on the expression of Otp mRNA in the entorhinal cortex of adult mouse, pig and human, using RNA-seq (see text footnote 3). However, our results provide high precision details on the distribution, morphology and some of the connections of these Otp cells. The most prominent output of the presubiculum is the bilateral projection to layer 3 of the medial entorhinal cortex (Caballero-Bleda and Witter, 1993; Witter and Amaral, 2004). Otp cells of the presubiculum may be engaged in such projections, which could contribute to the densely labeled neuropil found in layer 3 of the medial entorhinal cortex. Regarding Otp cells of the parasubiculum, these may project to the presubiculum and contribute to the densely labeled neuropil observed in layer 1 of this area. In addition, they may also project to other areas rich in labeled neuropil, such as the entorhinal cortex and the hippocampal formation (in accordance to the projections reviewed previously by Witter and Amaral, 2004). With respect to the Otp cells of the caudomedial entorhinal cortex (MEC), these cells appear to project to the hippocampal formation, based on the observation of labeled axons leaving this area and entering the deep white layer/subiculum transition zone, continuing rostrally in the alveus. Possibly these contribute to the varicose axons observed in the stratum oriens and in the stratum lacunosum moleculare (where the perforant pathway ends; Witter and Amaral., 2004). Some of the labeled fibers seen in the alveus cross the hippocampal commissure and might contribute to association between both hemispheres. In addition, some fibers might join the cingulate bundle and reach through it more anterior cortical areas, or subcortical regions, as described by Jones and Witter (2007); see also review by Bubb et al. (2018). Principal cells of MEC layer 2 include two chemical types, containing either calbindin or reelin (Ohara et al., 2019). The Otp-specific projection patterns of MEC suggested by us agree with those previously shown for the calbindin projection neurons, which are located in deep layer 2 and adjacent layer 3 of MEC (Ohara et al., 2019). In particular, these calbindin cells provide excitatory projections to the contralateral medial entorhinal cortex, to the stratum lacunosum of CA1, and to the septum (Ohara et al., 2019). However, the majority of the projections of these calbindin cells are intrinsic and target layer 2 of the MEC (Ohara et al., 2019), where we see a dense Otp-specific GFP-labeled neuropil. It appears that the calbindin neurons of MEC are heterogeneous, and also are strongly collateralized (Ohara et al., 2019). More studies are needed to investigate the precise molecular signature and connections of the Otp cells found in MEC, to know how they relate to the different calbindin cell subtypes and to other cells of MEC.

Another interesting aspect to remark is the ring-like organization of the Otp cells in the caudomedial entorhinal cortex, where the Otp cells surround a central area or island containing labeled processes but free of Otp somata. This organization suggests that peripheric Otp cells regulate activity of the central cells. The medial entorhinal cortex is a major interface for regulating spatial cognition, where grid, head-direction, and border cells coexist (Witter and Moser, 2006; Moser et al., 2008). It is interconnected with the hippocampal formation, and its complex microcircuit organization allows integration and computation of different signals to obtain a precise metric representation of position in space (Witter and Moser, 2006; Moser et al., 2008; Burgalossi et al., 2011). It contains small and large patches displaying cytochrome oxidase activity: small patches distribute modularly along layer 2 of the medial entorhinal cortex, and large patches are located close to the dorsomedial border, in an area classically defined as part of the parasubiculum (Caballero-Bleda and Witter, 1993; discussed by Burgalossi et al., 2011). Our results show that both areas contain Otp cells, but it is in relation to the small patches where Otp cells appear to organize around the center, although this requires confirmation by double labeling. Caudally, the large patch area appears to extend laterally along the dorsal border of the entorhinal cortex. Cells in large patches are head-direction selective, while those of the small patches are not head-direction selective and might represent attractor-like modules within a grid cell network (Witter and Moser, 2006; Burgalossi et al., 2011). Both patches are interconnected, and this might contribute to integration and computation processes needed to obtain a precise spatial map (Burgalossi et al., 2011). The Otp cells found in the large patch area, as well as around the small patches might participate in the regulation of these complex computations.

Finally, in contrast to the caudomedial entorhinal cortex, the lateral entorhinal cortex is free of Otp cells. This provides additional data for a distinction between lateral (rostrolateral) and medial (caudomedial) entorhinal cortex divisions, which would agree with the proposal that they derive from different embryonic compartments (Abellán et al., 2014; Medina et al., 2017, 2021).

## Data Availability Statement

The raw data supporting the conclusions of this article will be made available by the authors, without undue reservation.

## Ethics Statement

The animal study was reviewed and approved by Committees of Ethics for Animal Experimentation and Biosecurity of the University of Lleida and the Catalonian Government (Generalitat de Catalunya).

## Author Contributions

LMo processed all the embryonic and early postnatal Otp-eGFP brains (until P19), and some of the adult Otp-eGFP and Otp-Cre; Rpl22-HA brains and also analyzed the material, photographed, and prepared many figures (this material is part of her Ph.D. research project). AG-A processed most of the adult Otp-Cre; Rpl22-HA and Otp-eGFP brains (as part of her Ph.D. project) and also analyzed the material and obtained digital photographs. ED and LMe analyzed the material and obtained additional digital photographs. LMo and ED prepared the final figures of the manuscript. LMo and LMe produced the first draft of the manuscript. All authors revised the manuscript.

## Conflict of Interest

The authors declare that the research was conducted in the absence of any commercial or financial relationships that could be construed as a potential conflict of interest.

## Publisher’s Note

All claims expressed in this article are solely those of the authors and do not necessarily represent those of their affiliated organizations, or those of the publisher, the editors and the reviewers. Any product that may be evaluated in this article, or claim that may be made by its manufacturer, is not guaranteed or endorsed by the publisher.

## Footnotes

1
https://developingmouse.brain-map.org/

2
https://www.proteinatlas.org/about

3
https://www.proteinatlas.org/ENSG00000171540-OTP/brain

## Abbreviations

AAv: anterior amygdala ventral part
ACo: anterior cortical amygdalar area
AHi: amygdalohippocampal area
alv: alveus of the hippocampus
APir: amygdalopiriform transition area
ASt: amygdalo-striatal transition area
BL: basolateral amygdala
BLv: ventral part of the basolateral amygdala
BM: basomedial amygdala
CA1: field CA1 of the hippocampus
CA3: field CA3 of the hippocampus
cc: corpus callosum
Ce: central nucleus of the amygdala
Cg: cingulate cortex
Cl: claustrum
DEn: dorsal endopiriform nucleus
DG: dentate gyrus
Fr: frontal cortex
HF: Hippocampal formation
hpc: hippocampal commissure
LEC: rostrolateral entorhinal cortex
LMol: lacunosum moleculare layer of the hippocampus
LOT: nucleus of the lateral olfactory tract
Lv: ventral part of the lateral amygdala
Me: medial nucleus of the amygdala
MeA: anterior subnucleus of Me
MEC: caudomedial entorhinal cortex
MeP: posterior subnucleus of Me
MePD: dorsal subnucleus of MeP
MePV: ventral subnucleus of MeP
NCx: neocortex
OB: olfactory bulb
och: optic chiasm
Or: oriens layer of the hippocampus
ot: optic tract
PA: pallial amygdala
PaS: parasubiculum
PFC: prefrontal cortex
Pir: piriform cortex
PLCo: posterolateral cortical amygdalar area
PMCo: posteromedial cortical amygdalar area
PO: preoptic area
PRh: perirhinal cortex
PrS: presubiculum
Py: pyramidal cell layer of the hippocampus
Rad: radiatum layer of the hippocampus
Se: septum
STr, subiculum: transition area
SuPO: subpreoptic area (at the terminal prosomeric part of TOH)
TOH: telencephalon-opto-hypothalamic embryonic domain.
